# Ashbya Genome Database 3.0: a cross-species genome and transcriptome browser for yeast biologists

**DOI:** 10.1186/1471-2164-8-9

**Published:** 2007-01-09

**Authors:** Alexandre Gattiker, Riccarda Rischatsch, Philippe Demougin, Sylvia Voegeli, Fred S Dietrich, Peter Philippsen, Michael Primig

**Affiliations:** 1 Biozentrum & Swiss Institute of Bioinformatics, Klingelbergstrasse 50-70, CH-4056 Basel, Switzerland; 2 Duke University, Research Drive, Durham, NC 27710, USA

## Abstract

**Background:**

The Ashbya Genome Database (AGD) 3.0 is an innovative cross-species genome and transcriptome browser based on release 40 of the Ensembl developer environment.

**Description:**

AGD 3.0 provides information on 4726 protein-encoding loci and 293 non-coding RNA genes present in the genome of the filamentous fungus *Ashbya gossypii*. A synteny viewer depicts the chromosomal location and orientation of orthologous genes in the budding yeast *Saccharomyces cerevisiae*. Genome-wide expression profiling data obtained with high-density oligonucleotide microarrays (GeneChips) are available for nearly all currently annotated protein-coding loci in *A. gossypii *and *S. cerevisiae*.

**Conclusion:**

AGD 3.0 hence provides yeast- and genome biologists with comprehensive report pages including reliable DNA annotation, Gene Ontology terms associated with *S. cerevisiae *orthologues and RNA expression data as well as numerous links to external sources of information. The database is accessible at .

## Background

Approximately 90% of the genes present in the genomes of the filamentous fungus *Ashbya gossypii *and the budding yeast *Saccharomyces cerevisiae *were found to be orthologous and syntenic [1]. This degree of similarity was remarkable since both species display distinct morphologies as well as growth and differentiation properties. In the case of *Ashbya*, for example, there is no evidence for sexual reproduction although its genome contains the relevant complement of genes involved in yeast meiosis and spore development [2]. Budding and fission yeasts are key model organisms for high-throughput studies aiming at elucidating gene function by deletion analysis [3,4], expression profiling of conserved processes such as the mitotic cell cycle [5-7] and meiotic development [8-10] and by analysing protein localization [11] or protein-protein interaction patterns [12,13]. The data obtained in these species are believed to be useful for analysing networks and predicting gene function in yeasts and related organisms in the emerging field of systems biology [14]. Similar functional genomics and expression profiling experiments are currently being carried out using *A. gossypii *but there is currently no comprehensive source of information that provides cross-species coverage of high-throughput annotation, functional analysis and expression profiling data.

Technological progress in the field of DNA sequencing led to the production of a huge amount of information and spawned the development of appropriate bioinformatics tools for DNA data interpretation and analysis. The Ensembl project provides a comprehensive annotation database covering 19 species. Moreover, it enables software engineers to build standalone applications within the freely available Ensembl development environment [15]. The urgent need for a global and coherent approach to genome annotation has lead to the formation of the Gene Ontology (GO) consortium. GO develops a controlled and structured vocabulary (Ontology) for describing the process a gene product is involved in as well as its molecular function and sub-cellular localization [16]. This effort has yielded annotation information very useful for the categorization of genes across species and has proven particularly pertinent for microarray data interpretation ([17] and references therein).

This paper reports the release of Ashbya Genome Database 3.0, an innovative cross-species genome and transcriptome browser based on the Ensembl environment. The DNA annotation and high-density oligonucleotide microarray RNA expression data available via AGD are regularly updated to continue providing an excellent source of online information for yeast and genome biologists.

## Construction and content

AGD 3.0 was developed using the Ensembl Application Programming Interface (API) and base web code release 40 [18-20]. Further details on the AGD developer environment have been published previously [21].

AGD 3.0 provides highly reliable and in many cases manually verified DNA annotation data on 4726 protein-coding genes present in the genome of *A. gossypii*. GeneOntology annotation data associated with orthologues from *S. cerevisiae *are displayed in the *A. gossypii *gene report page. Whole-genome expression profiling data are displayed for 4451 annotated genes represented in the database and present on proprietary GeneChips used to study gene expression during *A. gossypii *spore germination (R. Rischatsch, P. Demougin, A. Gattiker, M. Primig and P. Philippsen, unpublished). A number of similar GeneChip-based transcriptome studies that cover mitotic growth [5] and meiotic development [10,22,23] in budding yeast have also been incorporated. This facilitates cross-species interpretation of transcriptional patterns and relative expression levels. Critically, the expression signals were obtained with the same robust microarray platform and experiments that are based on the same array architecture (e.g. *Saccharomyces *S98 and *Ashbya *SYNG001 GeneChips) and are therefore directly comparable even between species [24-26].

To analyze homology at the DNA and protein levels we used BLASTZ [27] and BLAT [28] as implemented in the Compara system [15]. Syntenic regions were computed by merging homology regions (identified by BLASTZ) that were in the same orientation and not more than 5000 bp apart in both organisms. Among the set of merged regions, only those covering at least 10000 bp were retained to ensure that syntenic regions contained a meaningful number of loci. Furthermore, complementary data from the Orthologous Matrix project (using a conservative approach to the identification of orthologs) were included to provide independent phylogenetic information on 287 species [29,30].

## Utility and discussion

The database can be searched by using a systematic or standard locus name (including aliases) from *A. gossypii *or *S. cerevisiae *via the welcome page. A wildcard option is provided (e.g. "CDC*"). Genes can also be searched through cross-references to GenBank/EMBL Protein identification numbers, UniProt accession numbers and Gene Ontology identifiers (e.g. "GO:0005737"). Finally, it is possible to retrieve genes or genome regions by DNA sequence coordinates.

The home page presents the *A. gossypii *genome as well as links to navigate to the *S. cerevisiae*, *S. pombe *and *N. crassa *genomes. The user can click on the karyotype or input sequence coordinates to display a specific chromosome. The *MapView *shows a summary display of the chromosome features. Clicking on a portion of the chromosome brings up the *ContigView *that shows features at three different scales. In the *DetailedView *section, pop-up menus can be used to display different features: genes, homologous regions from other fungal species mapped by BLASTZ and, notably, the positions of the target sequences covered by oligonucleotide probes on *Ashbya *and yeast GeneChips. Selecting the "View alongside..." option in the navigation menu brings up the *MultiContigView*, which displays homologous and syntenic (similarly oriented) genes from two species. The information in the gene pages has been substantially extended since the previous release [21]. Cross-references to Gene Ontology (GO, based on data supplied by SGD in *S. cerevisiae *homologs), GenBank/EMBL and UniProtKB have been added. Clicking on a GO term allows browsing all entries sharing a given annotation. Furthermore, links to facilitate information retrieval from external sources have been added. This includes the display of recently published articles from the Google Scholar service, and the display of community annotation entries from GermOnline [31,32].

**Figure 1:**
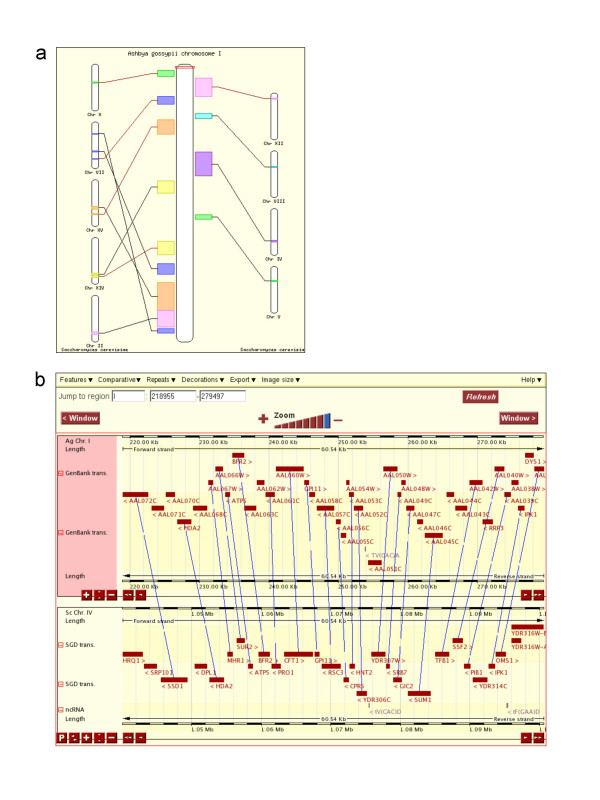
The synteny viewer. Panel a displays syntenic chromosomal regions from *A. gossypii *and *S. cerevisiae*. Panel b shows different levels of the *MultiContigView *as indicated where homologous genes in a given region are connected with lines.

The conservation of gene order across genomes can be visualized at two scales. The *SyntenyView *page is available from the chromosome page and displays locations of large-scale syntenic regions on the chromosomes of two selected species (Figure 1, panel a). The *MultiContigView *page can be accessed from the synteny view or from the Orthologue Prediction section of a gene page and displays the genomic maps of two organisms at a high resolution. In that view, homologous genes are linked by blue lines (panel b).

The gene report page contains a new section that displays putative orthologs of genes present in the *Ashbya *genome. Information from the Compara pipeline [15] and the Orthologous Matrix (OMA) [29,30] is summarized. While both methods start with an all-against-all Smith-Waterman protein similarity search the results are not identical because different algorithms are used. Compara data indicate best reciprocal BLASTP hits among the four loaded fungal genomes, indicating putative orthologs even with relatively low similarity. OMA data contains 287 complete genomes from all kingdoms and attempts to detect truly orthologous relationships.

**Figure 2:**
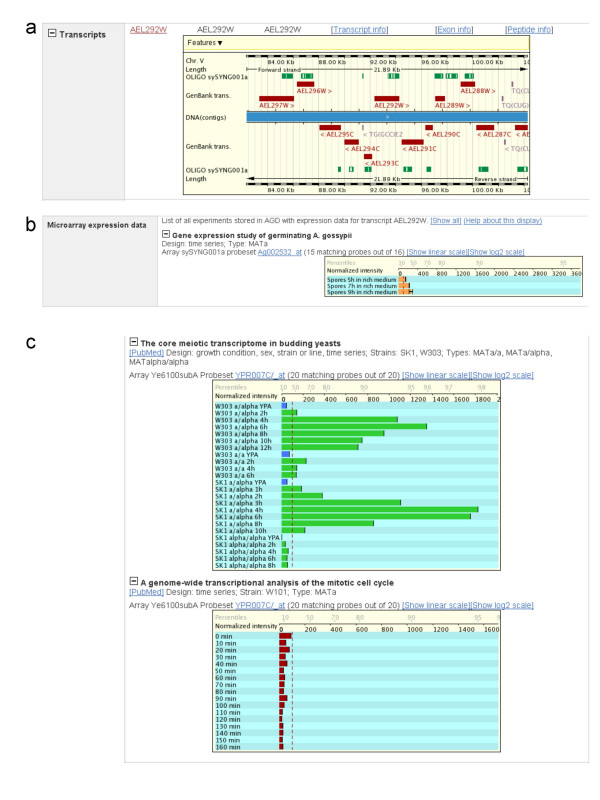
Microarray expression data for *A. gossypii *and *S. cerevisiae*. Panel a gives an overview of the genomic localization of genes. The target sequences recognized by microarray oligonucleotide probes present on the *Ashbya *GeneChip (SYNG001a) are indicated in green. Panels b and c show a bar diagram of normalized array expression signals obtained with germinating *Ashbya *spores and hyphae growing on rich medium compared to data obtained for the budding yeast orthologs expressed on various growth and sporulation media as indicated.

A section of the report page covers microarray expression data annotated using our Microarray Information Management and Annotation System (MIMAS) [33]. Users can view information on the array type, experimental details and a detailed sample history that is available in a popup menu via clickable sample names. Normalized microarray expression signals are displayed in the context of the DNA annotation (Figure 2, panel a) in a bar diagram as linear or log2-transformed values and the percentile of genes transcribed at the given level is indicated. Currently, expression levels are available from samples of germinating *Ashbya *somatic spores incubated in rich medium for five, seven and nine hours (panel b). The array data volume is expected to increase in size rapidly as results from ongoing research will constantly be added. By clicking on the appropriate links in the "orthologs prediction" section of the report page users can access comparable data available for mitotically growing and sporulating budding yeast (panel c). It should be noted that the expression signals obtained with microarrays in different species indicate relative but not absolute mRNA levels.

The sequence and annotation information is kept synchronized between AGD and the GenBank/EMBL/DDBJ collaboration [34]. The data are accessible in any of the databases under accession numbers AE016814 through AE016821. The 'Export data' link in AGD also allows creating custom files in a range of formats containing sequence data and/or feature annotation within a certain sequence range. Results of the microarray study using proprietary *Ashbya *GeneChips will be published elsewhere and the raw expression signal values will be made available via ArrayExpress, a certified public repository at the European Bioinformatics Institute [35].

Upcoming releases of AGD will contain information about gene function from large-scale deletion studies in *A. gossypii *and predicted protein-protein interaction data on the basis of high-throughput studies in *S. cerevisiae *[12,13]. Finally, high-density oligonucleotide microarray expression data from the species represented in AGD will continuously be integrated as they become available.

## Conclusion

AGD 3.0 is a useful and innovative database for researchers in the yeast and genome evolution fields. The gene annotation data is steadily verified and improved and it is now complemented with high-density oligonucleotide microarray expression data. This approach marks out AGD 3.0 as the first database that enables users to view a comprehensive report page including information on gene DNA annotation and RNA expression across conserved fungal species.

## Availability and requirements

Project name: Ashbya Genome Database

Project home page: 

Operating system(s): Linux

Programming language: Perl

Licence: the database is freely accessible for academic users under the GNU GPL.

Restrictions to use by non-academics: commercial users are referred to Syngenta for more details on access to microarray expression signals obtained with proprietary GeneChips.

Deep links into AGD from external sources are possible via the uniform resource locator (URL)  where [NAME] can be a budding yeast gene systematic name (e.g. *YPR175W*) or standard name (*SPO11*) or an *A. gossypii *systematic name (e.g. *AEL267C*).

## Authors' contributions

AG developed the database, RR contributed microarray expression data, PD participated in array data production, FSD participated in genome annotation and GeneChip development, PP lead the genome annotation project, MP participated in the development of AGD 3.0' concept and wrote the paper. All authors read and approved the final manuscript.
